# FA Polymerization Disruption by Protic Polar Solvents

**DOI:** 10.3390/polym10050529

**Published:** 2018-05-15

**Authors:** Guillaume Falco, Nathanaël Guigo, Luc Vincent, Nicolas Sbirrazzuoli

**Affiliations:** Institut de Chimie de Nice, Université Nice-Sophia Antipolis, Université Côte d’Azur, UMR CNRS 7272, Nice CEDEX 06108, France; guillaume.falco@insa-lyon.fr (G.F.); lvincent@unice.fr (L.V.)

**Keywords:** renewable resources, lignocellulosic biomass, polymerization, reaction mechanisms, furfuryl alcohol

## Abstract

Furfuryl alcohol (FA) is a biobased monomer derived from lignocellulosic biomass. The present work describes its polymerization in the presence of protic polar solvents, i.e., water or isopropyl alcohol (IPA), using maleic anhydride (MA) as an acidic initiator. The polymerization was followed from the liquid to the rubbery state by combining DSC and DMA data. In the liquid state, IPA disrupts the expected reactions during the FA polymerization due to a stabilization of the furfuryl carbenium center. This causes the initiation of the polymerization at a higher temperature, which is also reflected by a higher activation energy. In the water system, the MA opening allows the reaction to start at a lower temperature. A higher pre-exponential factor value is obtained in that case. The DMA study of the final branching reaction occurring in the rubbery state has highlighted a continuous increase of elastic modulus until 290 °C. This increasing tendency of modulus was exploited to obtain activation energy dependences (*E*_α_) of FA polymerization in the rubbery state.

## 1. Introduction

Lignocellulosic biomass appears as an important renewable source for the fabrication of organic polymeric materials [1,2,3]. In this line, furan derivatives [4] such as 5-hydroxymethylfurfural [5] or furfural [6] are respectively obtained from C_6_ or C_5_ sugar dehydrations [7] in the bio-refinery processes. These two compounds are among the most interesting biobased building blocks to design a sustainable chemistry [8] and they can be further transformed [9] in, for instance, 2,5-furandicarboxylic acid and furfuryl alcohol (FA), respectively. These latter compounds are indeed the top two of the class of biobased precursors used for their polymerization abilities.

FA is an attractive biobased compound which has the particularity to homopolymerize in poly(furfuryl alcohol) (PFA) under an acid catalyzed condition, leading to a strong reticulated network with high thermomechanical properties [10]. These advantages make the PFA an excellent biobased alternative in industrial processes such as for foundry molds [11] or wood reinforcement [12]. Other studies have demonstrated several applications for this eco-friendly polymer as the fabrication of carbon nanospheres [13], hybrid materials with silica [14,15], fully biobased composites with several natural fibers [16,17], or copolymers with a combination of vegetable oils [18,19].

**Figure 1:**
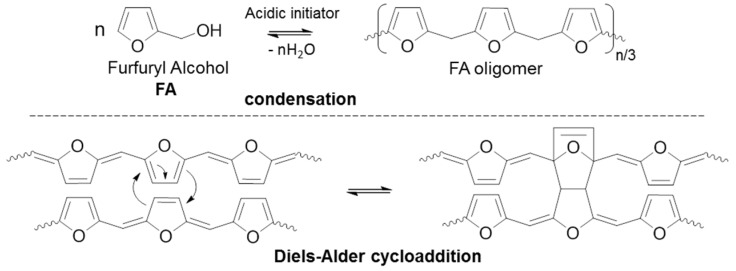
Polymerization mechanisms of FA.

As shown in Figure 1, the FA polymerization mechanism [10,20,21] is complex and can be divided into two steps. The first polymerization step occurs in the liquid phase with the influence of the acid initiator. This step consists of the formation of FA oligomers by polycondensation from an active furfuryl carbenium center. The second step leads to a three-dimensional branched polymer through Diels-Alder cycloadditions between the formed oligomers. Furthermore, side reactions can also occur. First, an electrophilic addition [22] of the active furfuryl carbenium center on the conjugate form of the oligomer can occur, increasing the heterogeneity of the final 3D network. Another reaction that was reported in the literature is FA furan ring opening [20], which preferably starts via C–O bond breaking [23]. Although spectroscopic [10,24] and theoretical [25] studies have highlighted the low percentage of furan ring-opening during the FA polymerization, these side reactions may have a significant impact on the final polymer structure. Indeed, furan ring openings lead to a lower final crosslinking density, impacting the mechanical properties of the final polymer [26]. Furthermore, these opened furan structures are amplified by the addition of protic polar solvents, suggesting disruption mechanisms of FA polymerization.

This paper aims to study the disruption effect of protic polar solvents during the polymerization of FA. Both water and isopropyl alcohol (IPA) were added as protic polar solvents in FA formulations with maleic anhydride (MA) as an acidic initiator. The polymerization evolution was followed from the liquid state by Differential Scanning Calorimetry (DSC) and additionally from the rubbery state by Dynamic Mechanical Analysis (DMA). The study provides, for the first time, an advanced isoconversional analysis of FA polymerization kinetics over a wide temperature range and including two stages of polymerization, i.e., an early liquid state and final rubbery state. The obtained variations of effective activation energy, *E*_α_, are interpreted as a function of the extent of conversion, α, and of the temperature. Important changes in the polymerization mechanism are discussed.

## 2. Materials and Methods

### 2.1. Materials

Furfuryl alcohol (FA) (*M*_w_ = 98.10 g·mol^−1^, b.p. = 170 °C, purity 99%), isopropyl alcohol (IPA) (*M*_w_ = 60.10 g·mol^−1^, b.p. = 82 °C, purity > 99.7%), and maleic anhydride (MA) (*M*_w_ = 98.06 g·mol^−1^, m.p. = 51–56 °C, purity > 99%) were obtained from Aldrich Chemical Co (Milwaukee, WI, USA).

### 2.2. Preparation of Liquid FA/solvent Mixtures

Three different liquid formulations containing FA, maleic anhydride (MA), and protic polar solvents were prepared for DSC investigation, namely: FA/MA (100/2); FA/MA/water; and FA/MA/IPA (50/1/50). MA was used as an acidic initiator of FA homopolymerization and added with 2% *w*/*w* compared to FA. For mixtures containing solvents, IPA or ultra-pure water (conductivity 2 µS·cm^−1^) was added to an equivalent quantity of FA (50/50 *w*/*w*) with 1% of MA to maintain the same initiator ratio as FA/MA.

### 2.3. Preparation of Cured PFA Materials

Three different reticulated resins were prepared from liquid formulations used in the DSC study, i.e., FA/MA (100/2); FA/MA/water; and FA/MA/IPA (50/1/50). Pre-polymers were prepared in a PTFE round-bottom flask by heating approximatively 15 grams of liquid formulations. The mixtures were vigorously stirred during the overall process to lead to homogenous final polymers. The synthesis of each furanic resin was realized in two steps. The first pre-polymerization step was the same for the three resins. Blends were heated at around 85 °C for one hour in a round-bottom flask. A condenser was used to avoid the evaporation of solvent or FA and to induce sufficient interactions between FA (or FA oligomers) and the protic polar solvent. For the second step, the condenser was removed and the resulting mixtures were placed at 100 °C. The temperature was increased by 10 °C every 30 min to obtain a highly viscous resin (~10^3^ Pa·s^−1^). To reach the desired viscosity, FA/MA and FA/MA/water were heated to 120 °C, while FA/MA/IPA was heated to 140 °C.

Then, the pre-polymers obtained were introduced in a silicon mold and were cured for two hours under pressure (~10 bars), respectively, at 160 °C for FA/MA and FA/MA/water and at 180 °C for FA/MA/IPA. This step was conducted to obtain rigid materials. The second curing step under pressure was essential to avoid the formation of holes in the PFA material (due to water evaporation by the polycondensation of FA), which can modify the mechanical or the thermal performances of the final materials.

After slow cooling to room temperature, polymers were unmolded and a post-curing step with a duration of one hour at 180 °C for FA/MA and FA/MA/water and at 220 °C for FA/MA/IPA was conducted in order to evaporate unreacted FA monomers and avoid possible trapped solvent. The three materials are labelled in the DMA study as follows: reference PFA (for PFA cured with pure FA), PFA/water (for PFA obtained via the water route), and PFA/IPA (for PFA obtained via the IPA route).

### 2.4. Analytical Techniques

Differential scanning calorimetry (DSC) measurements were performed on a Mettler-Toledo DSC-1(Mettler-Toledo GmbH, Schwerzenbach, Switzerland) equipped with a FRS5 sensor (with 56 thermocouples Au-Au/Pd, Mettler-Toledo GmbH, Schwerzenbach, Switzerland) and STAR© software (Mettler-Toledo GmbH, Schwerzenbach, Switzerland) for data analysis. Temperature and enthalpy calibrations were performed using indium and zinc standards. Samples of about 10 mg were placed into a sealed 30 μL high-pressure crucible. The DSC measurements of FA polymerization were conducted at the heating rates of 1, 2, 4, and 6 °C·min^−1^.

Dynamic mechanical properties were carried out on a Mettler-Toledo DMA 1 (Mettler-Toledo GmbH, Schwerzenbach, Switzerland) in tensile mode equipped with STAR© software for curves analysis. The sample dimensions were 15.00 (length), 4.50 mm (width), and 1.50 mm (thickness) (±0.01 mm). The experiments were performed from 25 to 350 °C, at a frequency of 1 Hz and with a heating of 1, 2, and 4 °C·min^−1^.

The DSC and DMA data were treated with an advanced isoconversional method to realize a kinetic study and to compute the activation energy dependency (*E*_α_) of FA polymerization. These computations were realized with internal software [27,28].

### 2.5. Theoretical Approaches

Isoconversional methods are amongst the more reliable kinetic methods for the treatment of thermoanalytical data, see, for example, [29,30,31,32]. The ICTAC Kinetics Committee has recommended the use of multiple temperature programs for the evaluation of reliable kinetic parameters [30]. The main advantages of isoconversional methods are that they afford an evaluation of the activation energy, *E*_α_, without assuming any particular form of the reaction model, *f*(α) or *g*(α), and that a change in the *E*_α_ variation, called *E_α_*-dependency, can generally be associated with a change in the reaction mechanism or in the rate-limiting step of the overall reaction rate, as measured with thermoanalytical techniques.

Polymerizations are frequently accompanied by a significant amount of heat released, thus cure kinetics can be easily monitored by DSC. It is generally assumed that the heat flow measured by calorimetry is proportional to the process rate [33]. Thus, the extent of conversion at time *t*, α_t_, is computed according to Equation (1), as follows:

(1) $$\alpha_{t}=\frac{\int_{t_{i}}^{t}\left(dQ/dt\right)dt}{\int_{t_{i}}^{t_{f}}\left(dQ/dt\right)dt}$$

where d*Q*/d*t* is the heat flow; *t_i_* is the time at which the process initiates (i.e., the respective heat flow becomes detectable); and *t_f_* is the time at which the process finishes (i.e., the heat flow falls below the detection limit). The denominator represents the total transformation heat released during curing (*Q*). 

In a more general sense, the extent of conversion can be determined as a change of any physical property associated with the reaction progress. For this, the physical property has to be normalized to lay between 0 and 1. If the shear modulus changes with the reaction progress, an extent of conversion can be defined as follows:

(2) $$\alpha_{t}=\frac{G_{t}^{\prime }-G_{t_{i}}^{\prime }}{G_{t_{f}}^{\prime }-G_{t_{i}}^{\prime }}$$

where *G*′ is the shear modulus measured by DMA (or dynamic rheometric) experiments at time *t*, and where *t_i_* and *t_f_* have the same meaning as in Equation (1).

The general form of the basic rate equation is usually written as [33,34]:

(3) $$\frac{d\alpha }{dt}=A\exp\left(\frac{-E}{RT}\right)f\left(\alpha \right)$$

where *T* is the temperature, *f*(α) is the differential form of the reaction model that represents the reaction mechanism, *E* is the activation energy, and *A* is the pre-exponential factor. 

The advanced isoconversional method [27,33,35,36] used in this study is presented in Equations (4) and (5) and has been derived from Equation (3), as follows:

(4) $$\Phi \left(E_{\alpha }\right)=\overset{n}{\underset{i=1}{\sum }}\overset{n}{\underset{j\neq 1}{\sum }}\frac{J\left[E_{\alpha },T_{i}\left(t_{\alpha }\right)\right]}{J\left[E_{\alpha },T_{J}\left(t_{\alpha }\right)\right]}$$

(5) $$J\left[E_{\alpha },T_{i}\left(t_{\alpha }\right)\right]=\overset{t_{\alpha }}{\underset{t_{\alpha -\Delta \alpha }}{\int }}\exp\left[\frac{-E_{\alpha }}{RT\left(t\right)}\right]dt$$

where *E_α_* is the effective activation energy. The *E*_α_ value is determined as the value that minimizes the function Φ (*E*_α_). This method is applicable to any arbitrary temperature program *T_i_*(*t*) and uses a numerical integration of the integral with respect to the time. *E*_α_ is computed for each value of α generally in the range 0.02–0.98 with a step of 0.02. For each *i*-th temperature program, the time *t_α_*_,_*_i_* and temperature *T_α_*_,_*_i_* related to selected values of α are determined by an accurate interpolation [27,28]. The software developed can treat any kind of isothermal or non-isothermal data from DSC, calorimetry (C80), TGA, DMA, or rheometry [27,28,37,38]. 

## 3. Results and Discussion

### 3.1. FA Polymerization Evolution from the Liquid State

#### 3.1.1. Non-Isothermal DSC Investigation

**Figure 2:**
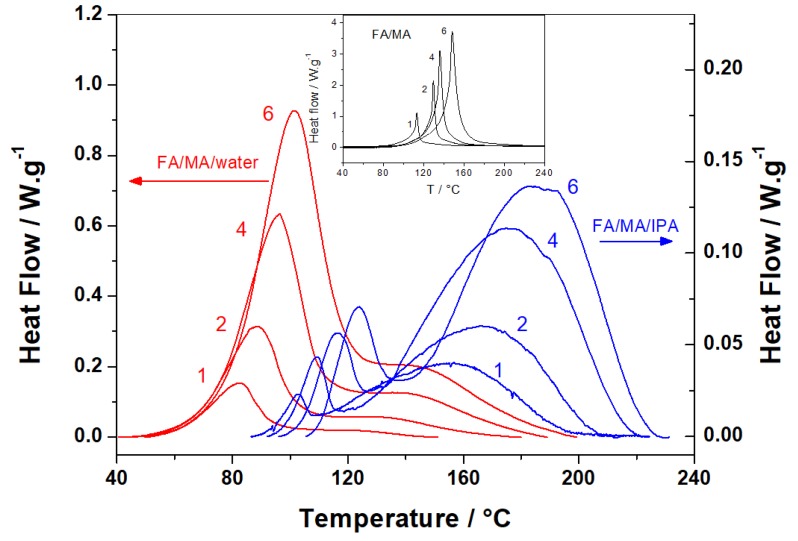
Non-isothermal DSC curves for the curing of FA/MA/water (red lines) and FA/MA/IPA (blue lines). Insert: FA/MA cure without solvent. The heating rates in °C·min^−1^ are indicated by the curves.

Figure 2 shows non-isothermal DSC data obtained during FA polymerization and in the presence of protic polar solvents such as water and IPA. The data of polymerization without solvent are presented in the insert of Figure 2 for comparison (reference system). The reference system shows a single asymmetric thermal event, while the addition of solvents significantly modifies the heat flow curve shapes, with the appearance of a shoulder or a second peak. This observation leads to the hypothesis of a change in the polymerization pathways. In the FA/MA/water mix, the first peak is predominant. It is followed by a broad thermal event that could correspond to secondary reactions or residual cross-links between FA oligomers. For the FA/MA/IPA mix, the polymerization pathway seems different from the other two systems due to a second and predominant thermal event. Moreover, the FA polymerization reactions are shifted to a higher temperature. The protic character of IPA due to its high dipolar moment (µ_IPA_ = 1.70 D) can explain this. The first step of FA polymerization starts with the formation of an active furfuryl carbenium center [10]. Thus, in this system, IPA slows down the formation rate of the carbenium center by forming hydrogen bonds and a solvation sphere with the hydroxyl groups of FA [39]. These effects could also occur in the presence of water, which also presents a high dipolar moment (µ_water_ = 1.85 D). However, the fact that MA is opened in maleic acid in the presence of water should also be taken into account. The pKa_1_ of maleic acid is very low (~1.8) and decreases the pH of the mix. When the FA polymerization is initiated in an acid catalyzed condition, the MA opening releases protons that allow the condensations to start at a lower temperature. Contrary to IPA, the slowdown effect due to solvation is counterbalanced by the formation of H^+^ due to the MA hydrolysis, which results in earlier initiation of FA polymerization with water.

These curves have been used to estimate the reaction heat released during the reaction (*Q*) by the integration of DSC peaks. The reaction heat values obtained for FA/MA/water and FA/MA/IPA systems are summarized and compared to the FA reference system [40] (Table 1). In order to compare values obtained from each formulation, the data were reported as the mass of FA. FA/MA/IPA systems show decreasing reaction heat values with an increasing heating rate. The values of the reaction heat are 2.4 to 3.5 times smaller than the reference values. Moreover, the reaction heat (*Q*) depends on the amplitude of the first thermal event, which is rather associated with initiation reactions. Indeed, increasing the heating rate allows less time for initiation reactions to take place and leads to a decrease of the reaction heat. These decreasing values, combined with the particular thermal events of this mix (two distinct peaks), suggest possible interactions between FA and IPA.

In the case of the FA/MA/water system, the opposite tendency occurs. The reaction heat increases with the heating rate. On the other hand, the highest reaction heat value of the FA/MA/water mix (712 g·mol^−1^) obtained for the fastest rate is almost the same as the reaction heat value of the FA/MA reference mix at the slower rate (709 g·mol^−1^). Thus, the heating rate considerably affects the reaction mechanism, which confirms the assumption of a complex multi-step polymerization mechanism. This also indicates that the final properties of the materials will be completely different according to the temperature domain used for curing the system.

#### 3.1.2. E_α_ vs. α-Dependence

**Figure 3:**
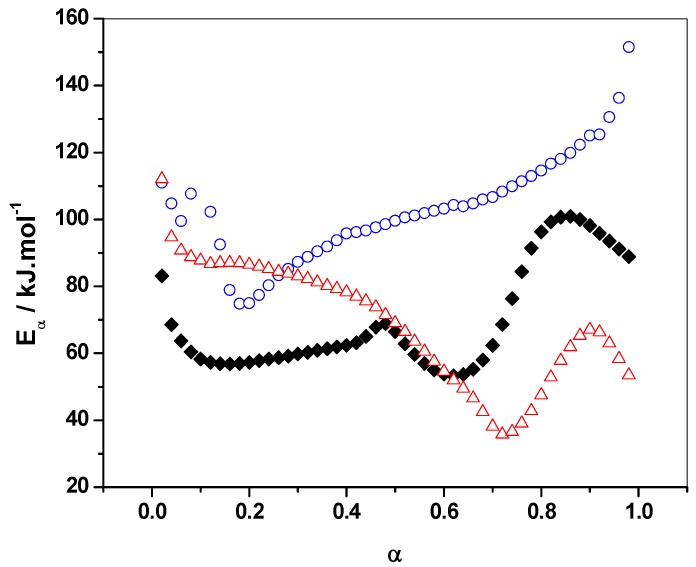
Dependence of the effective activation energy (*E*_α_) on extent of conversion of FA/MA/water (open red triangles), FA/MA/IPA (open blue circles), and FA/MA (solid black lozenges).

Model-free advanced isoconversional analysis was employed to highlight new insights on the complex polymerization mechanism and kinetics in the presence of solvents. Figure 3 represents the *E*_α_ dependencies with the extent of conversion (α). Analysis of the *E*_α_-dependencies clearly indicates a complex mechanism that involves several chemical steps, each of them having their own activation energy. As a result, each increasing and decreasing part of the effective activation energy (*E*_α_) can be associated with changes in the rate-limiting steps of the overall polymerization.

The three systems exhibit decreasing *E_α_* values in the initial stages of the reaction (for α values, until 0.10 for reference and water systems, and 0.20 for the IPA system) that can be attributed to an autocatalytic step [41]. This initial step corresponds to the formation of an active furfuryl carbenium center that will induce the polymerization. The longer decay of FA/MA/IPA *E*_α_ values to a higher extent of conversion (0.20) confirms the hypothesis of interactions between FA and IPA by hydrogen bonds or solvation spheres, which slow down the autocatalytic step.

The FA disruption polymerization by IPA is also confirmed by the completely different activation energy dependency obtained compared to the two other systems. Indeed, while alternating decreasing and increasing values are obtained for FA/MA and FA/MA/water, the FA/MA/IPA system reveals a continuous activation energy increase for α values from 0.20 to 1. The progressive increase observed might correspond to competitive or consecutive reactions (i.e., condensation, Diels-Alder and possible side reactions) during the polymerization [27]. On the other hand, *E*_α_-dependencies of FA/MA/water and reference systems show several similarities. The polycondensation of FA occurs for α ≈ 0.10 to α ≈ 0.40 for the two systems. A slight *E*_α_ decrease for FA/MA/water and a slight increase for the reference system characterize this step. The presence of water decreases the viscosity, which facilitates the polymerization of FA.

For α ≈ 0.48, *E*_α_ values of both systems are very close and a slight increase in the activation energy is observed for the reference system. It has been demonstrated that this increasing tendency for the reference system is correlated with the high viscosity increase due to the formation of crosslinks [40]. This phenomenon is not visible for FA/MA/water due to the presence of water, which still induces a lower viscosity at the same extent of conversion.

For α > 0.48, the decreasing tendency for the two curves is still the same, with a slight shift to a higher extent of conversion for the FA/MA/water system. At this stage of the reaction, the molecular mobility strongly decreases, which induces a decrease of the reaction rate. Thus, the overall polymerization becomes controlled by the diffusion of short linear chains. This is mainly due to an increase of the viscosity of the system, which reduces the chemical reactions rates and leads to a decrease of *E*_α_ [41]. This decrease is observed for both systems and is characteristic of a transition from a kinetic to diffusion regime [40]. Diffusion control generally becomes rate limiting when the characteristic relaxation time of the reaction medium markedly exceeds the characteristic time of the reaction itself [29]. This decrease to low *E*_α_ values is less marked, but is also present for the FA/MA system (*E*_α_ decreasing from 69 to 53 kJ·mol^−1^ for α from 0.48 to 0.63) than FA/MA/water (*E*_α_ decreasing from 71 to 36 kJ·mol^−1^ for α from 0.48 to 0.73). This indicates that diffusion control is more important in the presence of water. This will be explained by an analysis of the dependence of the effective activation energy (*E*_α_) on temperature (*T*).

Following this decrease, an increasing *E_α_*-dependency is observed for both FA/MA (until α ≈ 0.90) and FA/MA/water (until α ≈ 0.85) systems and corresponds to an increase in molecular mobility due to temperature increase. This mobility increase permits the chemical reaction to be reactivated and corresponds to the formation of chemical bonds in the gelled state by Diels-Alder cycloadditions. Finally, the last decrease is attributed to the diffusion of unreacted FA monomers [40].

According to Figure 3, *E*_α_ for FA/MA/water is always higher than *E*_α_ for FA/MA for 0 < α < 0.60, while the reaction in the presence of water is shifted to lower temperatures (Figure 2). Generally, a higher activation energy shifts the reaction to a higher temperature. Thus, in this case, it seems that the opposite effect is obtained. Because *E* and *A* have opposite effects regarding the shift of a reaction to a lower or higher temperature, this observation could be explained by a higher value of the pre-exponential factor for the FA/MA/water system. In order to verify this, pre-exponential factors were computed for FA/MA and FA/MA/water systems using the model-free method explained in detail in ref. [27]. The method uses the so-called false compensation effect that allows one to establish a relationship between *E*_α_ and ln*A*_α_ in the form: ln*A*_α_ = *aE*_α_ + *b* and is based on the practical observation that for complex (multi-step) processes, the same experimental curve can be described by several reaction models. Once this relation has been established, it is possible to compute ln*A*_α_ in a model-free way using the value of *E*_α_ obtained for each α value with an advanced isoconversional method. The computations were realized for a heating rate of 2 °C·min^−1^ and an extent of conversion 0.05 < α < 0.15. This range was selected in order to compute the pre-exponential factors at the very beginning of the reaction. The models that lead to the best fit (*r*^2^ > 0.9995) were the model numbers 4, 5, 6, 10, 11, 12, and 13 of ref. [27]. The parameters found for FA/MA are *a* = 0.29475 mol·kJ^−1^ and *b* = −6.47597 (*r*^2^ = 0.9984), and for FA/MA/water, they are a = 0.32955 mol·kJ^−1^ and *b* = −6.23381 (*r*^2^ = 0.9987). These values confirm our hypothesis that much higher values for the pre-exponential factor are obtained in the presence of water. As an example, the pre-exponential factors obtained for FA/MA and FA/MA/water for α = 0.10 are 4.48 × 10^4^ s^−1^ and 7.23 × 10^9^ s^−1^, respectively.

#### 3.1.3. Additionnal Kinetic Computation of FA/MA/IPA

**Figure 4:**
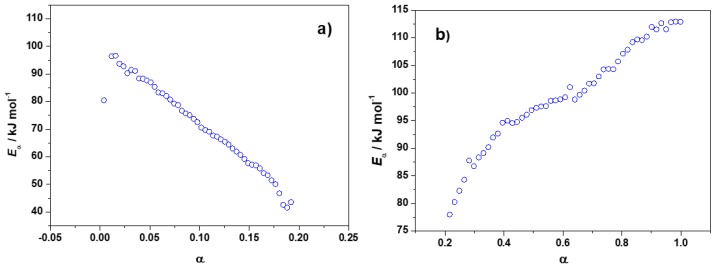
Partial dependence of the effective activation energy (*E*_α_) calculated from the non-isothermal DSC curves of FA/MA/IPA mix of Figure 2. (**a**) from the first thermal event (before 120/140 °C) and (**b**) from the second thermal event (after 120/140 °C).

To better understand the complex reactivity of FA/MA/IPA, additional computations were performed for each thermal event of this system (see Figure 2). Thus, the *E*_α_ calculated from the first thermal event (before 120/140 °C) is shown in Figure 4a, while Figure 4b shows the *E*_α_ calculated from the second thermal event (after 120/140 °C). It can be seen that the activation energy of Figure 4a has the same tendency as the curve of Figure 3 from α = 0 to α = 0.20, with a continuous *E*_α_ decrease. Therefore, the first thermal event of the FA/MA/IPA thermograms of Figure 2 is the result of the autocatalytic step. The second thermal event could be related to competitive reactions during the polymerization. These competitive reactions are displayed in Figure 4b, where a continuous increase of *E*_α_ for α from 0.20 to 1 is observed.

#### 3.1.4. E_α_ vs. T-Dependence

**Figure 5:**
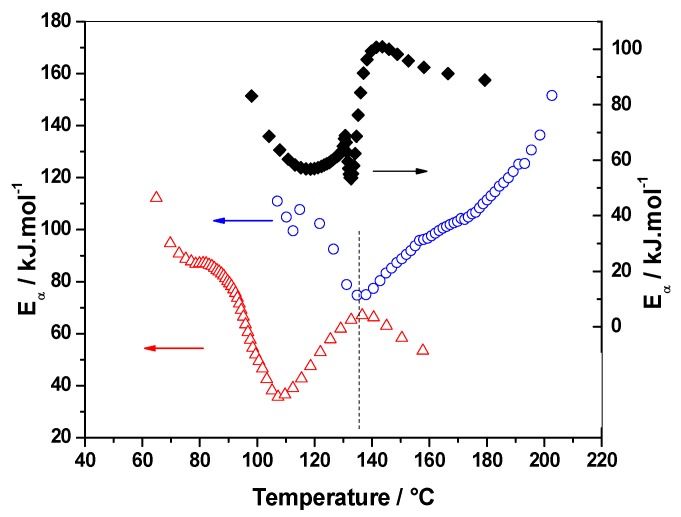
Dependence of the effective activation energy (*E*_α_) on temperature (*T*) for non-isothermal curing of FA/MA/water (open red triangles), FA/MA (solid black lozenges), and FA/MA/IPA (open blue circles) systems.

The apparent activation energy can also be computed as a function of temperature (*E*_α_–*T* dependency), by taking an average temperature associated with each value of the extent of conversion. Figure 5 represents the *E*_α_-dependencies with temperature. Analysis of *E*_α_–*T* values shows that the various rate-limiting steps are extended over a higher temperature range for the solvent systems and each of these steps takes place at different temperatures depending on the polymerization environment of FA. More precisely, Figure 5 highlights that the reaction of the FA/MA/water system is shifted to lower temperatures and the reaction of FA/MA/IPA to higher temperatures compared to the reference.

Indeed, the first *E_α_* decrease associated with the autocatalytic step occurs between 100–115 °C for FA/MA, 60–75 °C for FA/MA/water, and 120–140 °C for FA/MA/IPA system. The shift to lower temperatures observed for the water mix system is explained by the easier MA opening into maleic acid due to the presence of water and correlates with the higher pre-exponential factor values previously reported. For FA/MA/IPA, analysis of the *E*_α_–*T* dependency confirms the higher activation energy barrier for the initial stages of the polymerization of FA in the presence of IPA instead of water. Furthermore, the polymerization reactions (i.e., condensation and Diels-Alder reactions) of this mix begin at 135 °C, i.e., when the polymerization is finished or almost finished for the two other formulations. This confirms our previous hypothesis that the solvation sphere formed between FA and IPA is very stable and hinders the reactions.

Another significant difference highlighted by the analysis of the *E*_α_–*T* dependency is the secondary *E*_α_ decreasing values due to the diffusion regime. This stage is observed in a very short temperature range for FA/MA (131–133 °C), while this step takes place over a wider temperature range for FA/MA/water (90–105 °C). For the FA/MA/IPA system, this stage is absent and *E_α_* values exhibit the same tendency as in Figure 3, where the major part of the FA/MA/IPA dependency demonstrates increasing *E*_α_ values. This *E*_α_ decrease characteristic of the diffusion control of small molecules is more pronounced for the FA/MA/water system (86 to 36 kJ·mol^−1^) than for the FA/MA system (69 to 54 kJ·mol^−1^), in agreement with the results of Figure 3. This could seem to be, a priori, contradictory. Nevertheless, analysis of the *E*_α_–*T* dependency shows that this decrease occurs at much lower temperatures for the FA/MA/water system. This explains that diffusion control is more pronounced in the presence of water due to the inferior molecular mobility at a lower temperature.

After the second decreasing step observed for FA/MA and FA/MA/water, the *E_α_* values re-increase significantly in a very sharp temperature interval (133–141 °C) for the reference system. This is attributed to the reactivation of chemical reactions and diffusion of long segments of the polymer chains. A similar increase is observed for the water system, but at a lower temperature and in a wider temperature interval (107–136 °C). This final re-increase was attributed to the cross-links formation in the gelled state due to Diels-Alder cycloadditions [40]. Thus, the addition of water allows for the re-activation of chemical reactions at a lower temperature, probably because of the higher mobility of the system.

### 3.2. Residual Cross-Linking Reactions in Rubbery State

#### 3.2.1. Non-Isothermal DMA Investigation

**Figure 6:**
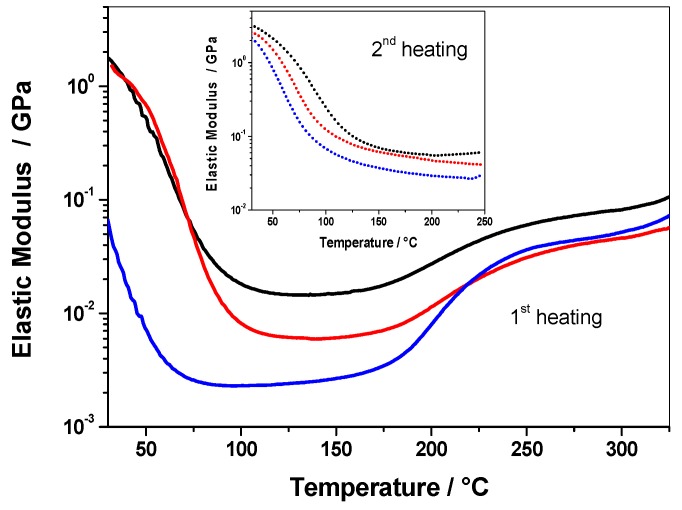
Elastic modulus vs. temperature obtained during the first heating (line) or the second heating (dot) at 2 °C·min^−1^ of reference PFA (black line), PFA/water (red line), and PFA/IPA (blue line).

Once polymerized, the mechanical properties of the three PFAs were evaluated by DMA. Elastic moduli of the three materials measured during the first heating scan or during the second scan (after a first heating to 250 °C) are shown in Figure 6. The decreasing moduli of about two decades between 30 and 130 °C for all the systems during the first scan are unambiguously attributed to the cooperative α-relaxation process of PFA chains commonly associated with the glass transition. Above 170 °C, i.e., from temperature range corresponding to the post-curing treatment, the elastic modulus increases for the three samples. This increase may correspond to residual cross-links occurring in solid PFA resins. As shown in Figure 6, the increase is more important when the PFA has been cured in the presence of protic polar solvents (e.g. water or IPA). In particular, it can be deduced that polymerization via IPA is less complete since the re-increase is about one decade and accordingly the amount of residual cross-links is more important compared to the other systems. Compared to the first scans, the second DMA scans measured after a first heating to 250 °C both show higher moduli and higher glass transition temperatures for all the samples. This indicates that the re-increase of moduli above 170 °C during the first scan can be attributed to residual crosslinks, thus increasing the crosslink density and consequently the chain mobility in PFA. It is worth noting that PFA/IPA and PFA/water shows lower glass transition temperatures and lower values of moduli compared to PFA. These features were attributed to furan ring opening reactions that are exacerbated during polymerization with protic polar solvents, thus leading to a lower cross-link density due to these more mobile entities [26]. 

**Figure 7:**
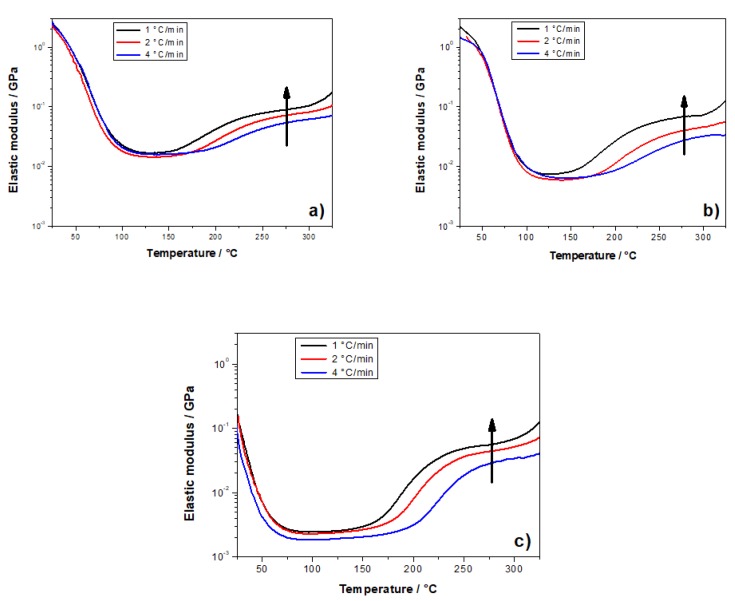
(**a**) Elastic modulus (*E*’) of three PFA samples obtained by DMA at 1, 2, and 4 °C. (**a**) reference PFA, (**b**) PFA/water, and (**c**) PFA/IPA.

Additional dynamic mechanical measurements were carried out at 1 and 4 °C·min^−1^ on each polymer from 25 to 350 °C (Figure 7) in order to further understand the kinetic of residual cross-links occurring in solid PFA resins. These DMA measurements performed at various heating rates were conducted to show that this increase is not due to the volatilization of either protic polar solvent added in formulations or water from FA polycondensations, which could remain trapped within polymer chains. These curves show that the moduli increase depends on the heating rate. Therefore, it can be deduced that it should be a kinetic phenomenon and not a thermodynamic phenomenon, such as a solvent evaporation. Indeed, first-order thermodynamic transitions such as vaporization should be independent of the heating rate. It is interesting to note that the modulus increase is observed from 170°C to 290 °C for the three systems in DMA, while no thermal event was recorded at a temperature higher than 220 °C on the first DSC heating curves. This clearly highlights the interest of using DMA measurements to identify residual crosslinks occurring in the rubbery state after intensive post-curing.

Then, the modulus increase above 150 °C clearly indicates that chemical reactions still occur in the rubbery state in the different PFA samples. The modulus increase is more important when the samples have been cured in the presence of protic polar solvents (e.g. water or IPA). More precisely, the polymerization in the presence of IPA is less complete since the re-increase and accordingly the amount of residual cross-links is more important compared to the other systems.

#### 3.2.2. E_α_ vs. T-Dependence

The kinetic of formation of these residual cross-links in the rubbery state was evaluated from the treatment of the DMA curves of Figure 7. For that purpose, the elastic modulus was normalized between 0 and 1 according to Equation (2), in order to get an extent of conversion, α, for the cross-links in the rubbery state. These data, obtained on the three PFA samples, were computed with an advanced isoconversional method (Equations (5) and (6)).

**Figure 8:**
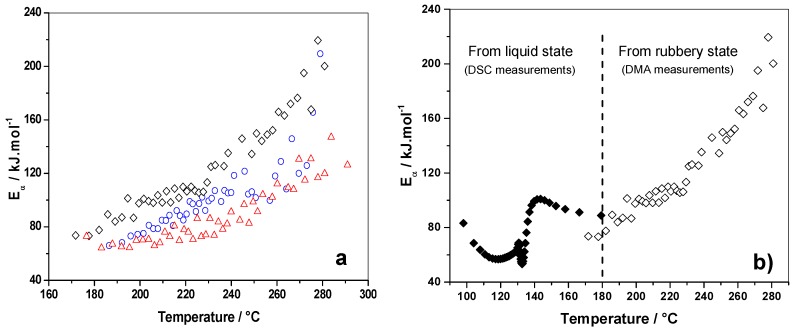
(**a**) Effective activation energy dependency (*E*_α_) as a function of temperature obtained from DMA measurements of Figure 7 (black open lozenges: PFA/MA, red open triangles: PFA/MA/water, blue open circles: PFA/MA/IPA); (**b**) Continuous *E*_α_ from liquid state (DSC measurements) to solid state (DMA measurements) for the FA/MA system.

Figure 8a shows the *E*_α_ dependencies computed as a function of temperature. The three samples demonstrate increasing *E*_α_ values, which could correspond to a kinetic control by the diffusion of long polymeric chains. As the PFA material progressively cross-links in the solid state, the molecular mobility becomes more restricted, which leads to an increase in the activation energy (*E*_α_). The PFA cured without solvent (i.e., PFA/MA, black lozenges) has the highest *E*_α_ values, which indicates that the long chains are more constrained in comparison to the PFA samples cured in the presence of solvents.

The *E*_α_–dependencies of the reference system obtained for polymerization from the liquid state (DSC measurements) and for additional cross-links occurring in the rubbery state (DMA measurements) were plotted in Figure 8b. As can be seen, there is a perfect continuity between the two dependencies, with the final values of the reaction starting from the liquid state being in good agreement with the first values of the reaction starting from the rubbery state. Thus, the combination of DSC and DMA data allows one to study the polymerization kinetics and to get mechanistic information over a wide temperature range, starting from the liquid state and ending in the solid state.

The modification of FA polymerization by the protic polar solvents (see Figure 8a) gives lower *E*_α_ values for reactions continuing in the rubbery state. The occurrence of ring opening reactions reduces the cross-link density [26] and thus, molecular mobility in the rubbery state is promoted. This result is in agreement with the lower values of the elastic modulus (*E*’) in the rubbery state for the PFA samples prepared with solvents (Figure 6). Absolute *E*_α_ values of PFA/water and PFA/IPA cannot be directly compared because these two samples were subjected to different post-curing treatments in order to obtain a sufficiently cohesive material for permitting DMA measurements. Nevertheless, the values are in the same order of magnitude.

## 4. Conclusions

The presence of solvent significantly disturbs the polymerization kinetics of FA. The autocatalytic stage is modified, likely due to a stabilization of the furfuryl carbenium center by either water or IPA. In the presence of water, the autocatalytic stage is less marked as the initiator, maleic anhydride, is rapidly hydrolyzed into maleic acid, which induces the onset of polymerization reactions at lower temperatures. In this mix, we note that FA polymerization rate-limiting steps are similar to the reference system, with only few variations of *E*_α_ values and temperatures of reaction. However, the presence of IPA shifts the beginning of the polymerization to a higher temperature. Furthermore, *E*_α_ values of FA/MA/IPA clearly indicate that main reactions and other side reactions seem to occur in parallel and throughout the whole polymerization. The only similarity between the *E*_α_ dependency of FA/MA/IPA and the two other systems is the autocatalytic step, which is characterized by a separate thermal event. 

The DMA study has highlighted a continuous increase of the elastic modulus attributed to further cross-links in the rubbery state until 290 °C. These additional reactions occurring in the rubbery plateau cannot be highlighted by DSC since the potential exothermic variation is too small to be detected. The *E*’ variations obtained for different heating rates permitted us to obtain *E*_α_-dependency in the rubbery state. It was shown that the modification of FA polymerization by protic polar solvents slightly decreases *E*_α_ values in the rubbery state due to the increase of molecular mobility. For reference PFA, the *E*_α_-dependencies obtained from the liquid state and from the rubbery state are in perfect continuity, which prove the validity of the new approach proposed to obtain mechanistic information over a very wide temperature range for complex polymerization. 

## Figures and Tables

**Table 1:** Reaction heat (*Q*) reported to the mass of FA for the three different systems: FA/MA/water, FA/MA/IPA, and FA/MA.

	FA/MA/Water	FA/MA/IPA	FA/MA (Reference)
β/°C·min^−^^1^	*Q*/J·g^−^^1^ of FA		
1	490 ± 20	300 ± 30	709 ± 30
2	632 ± 30	230 ± 20	685 ± 30
4	678 ± 30	216 ± 20	620 ± 30
6	712 ± 30	168 ± 20	593 ± 30
